# High Prevalence of *Prototheca bovis* Infection in Dairy Cattle with Chronic Mastitis in Ecuador

**DOI:** 10.3390/vetsci9120659

**Published:** 2022-11-25

**Authors:** María P. Huilca-Ibarra, David Vasco-Julio, Yanua Ledesma, Salome Guerrero-Freire, Jeannete Zurita, Pablo Castillejo, Francisco Barceló Blasco, Lisseth Yanez, Darwin Changoluisa, Gustavo Echeverría, Carlos Bastidas-Caldes, Jacobus H. de Waard

**Affiliations:** 1 Facultad de Ingenierías y Ciencias Aplicadas, Ingeniería en Biotecnología, Universidad de Las Américas, Quito 170124, Ecuador; 2 Unidad de Investigación en Biomedicina, Zurita & Zurita Laboratorios, Quito 170104, Ecuador; 3 Grupo de Investigación en Biodiversidad, Medio Ambiente y Salud (BIOMAS), Universidad de las Américas, Quito 170124, Ecuador; 4 Instituto de Investigación en Salud Pública y Zoonosis-CIZ, Universidad Central del Ecuador, Quito 170521, Ecuador; 5 Programa de Doctorado en Salud Pública y Animal, Facultad de Veterinaria, Universidad de Extremadura, 10003 Cáceres, Spain; 6 One Health Research Group, Facultad de Ciencias de la Salud, Universidad de Las Américas, Quito 170124, Ecuador

**Keywords:** *Prototheca bovis*, bovine mastitis, microalga, cattle, Ecuador, prevalence

## Abstract

**Simple Summary:**

*Prototheca bovis* is a non-photosynthetic alga ubiquitously distributed in water, sewage and soil. The alga appears to be responsible for a significant proportion of bovine mastitis cases in some countries, and occurs worldwide in tropical and temperate climatic areas. To date, no effective and economically favorable treatment is available, and control therefore requires culling of the infected animals. In the present study, we isolated *P. bovis* from 15.1% of cows with chronic mastitis from a tropical region in Ecuador. Species identification was carried out by nucleotide sequence analysis of the cytochrome b (*cytB*) gene. This is the first report that confirms the presence of *P. bovis* in cattle with mastitis in Ecuador.

**Abstract:**

The genus *Prototheca*, a unicellular, non-photosynthetic, yeast-like microalgae, is a pathogen of concern for the dairy industry. It causes bovine mastitis that currently cannot be cured, and hence generates significant economic losses in milk production. In this study, for the first time in Ecuador, we identify *Prototheca bovis* as the etiologic agent of chronic mastitis in dairy cattle. Milk samples (*n* = 458) of cows with chronic mastitis were cultured on Sabouraud Dextrose Agar (SDA). Microscopy and *cytB* gene sequencing were used to identify *Prototheca,* whereby *Prototheca bovis* was isolated from 15.1% (*n* = 69) of the milk samples, one of the highest infection rates that can be found in the literature in a “non-outbreak” situation. No other *Prototheca* species were found. We were unable to isolate the alga from environmental samples. We showed that *P. bovis* was relatively resistant to disinfectants used to sterilize milking equipment on the cattle farms where it was isolated. We discuss how to avoid future infection and also hypothesize that the real prevalence of *Prototheca* infection in bovine mastitis is probably much higher than what was detected. We recommend a protocol to increase the diagnostic yield in the bacteriology laboratory.

## 1. Introduction

*Prototheca* is a genus of algae in the family Chlorellaceae and can be found in soil and aqueous environments. They are unicellular organisms that lack chlorophyll and are capable of producing a cutaneous or invasive disease, called Protothecosis, in humans and other mammals [1,2]. *Chlorella*, green algae with chlorophyll, is the only other genus of algae that has been reported to cause invasive disease in humans and animals [3]. 

One of the reasons *Prototheca* is an opportunistic infectious agent is that with the loss of photosynthetic activity, these microorganisms need to adapt to heterotrophic conditions. Concerning its virulence, *Prototheca* generally shows its pathogenicity only under specific conditions in hosts with weakened immune responses [4]. 

Within the genus *Prototheca*, there are 15 species, of which *P. zopfii* genotype 1 and 2 (recently renamed respectively *P. ciferri* and *P. bovis*)*,* and *P. wickerhamii, P. cutis,* and *P. blaschkeae* have been related to infections in both animals and humans [5,6,7,8]. *P*. *bovis* and *P*. *wickerhamii* maintain the largest host ranges, including cats, dogs, buffaloes, horses (*P*. *bovis* only), and goats (*P*. *wickerhamii* only). *P. wickerhamii* and *P. cutis* are the most important species that infect humans, especially immunocompromised patients, and can cause peritonitis, septicemia, and meningitis [9,10,11]. However, recently a *P. zopffi* variant usually only found in animal infections was described in a bloodstream infection in an immunocompromised patient [12]. 

Infections of *Prototheca* in cattle typically present as mastitis and were first reported by Lerche in 1952 [13]. Ever since then, bovine mastitis due to *Prototheca* has been reported throughout all continents, and *P. bovis* is usually the infectious agent, followed by *P. blaschkeae* [14]. Infection of the mammary tissue can be subclinical: it is only detectable through raised somatic cell counts and the detection of *Prototheca* in the milk. Subclinical mastitis does not have visual signs. Acute or chronic mastitis due to this alga causes visibly abnormal milk, often swelling of the mammary gland and reduced milk yield. The prevalence of *Prototheca* spp. in routine milk samples for the isolation of mastitis pathogens is generally very low (0.1% of the submitted samples) [15]. However, if left uncontrolled, the infection can spread through the entire herd [8,16], causing great economic losses to the dairy farm. In outbreak situations, Protothecosis can affect more than 30% of the lactating herd [15,16,17].

Diagnosing infection caused by *Prototheca* spp., reported in a growing number of cattle herds around the world in recent decades, is important as there is no treatment for this infection. Cows should be culled as soon as the infection has been confirmed, in order to prevent the infection from spreading to other cows and to avoid unnecessary antibiotic use [18,19]. In Ecuador, mastitis rarely has a microbiological diagnosis as this is too expensive, especially for smaller cattle farms. In addition, only a few professional laboratories are able to perform this type of microbiological analysis. The aim of this study was to isolate *Prototheca* as a cause of mastitis in dairy in Ecuador and to identify the *Prototheca* specie(s) involved. 

## 2. Materials and Methods

### 2.1. Study Area, Population, and Sampling

**Figure 1:**
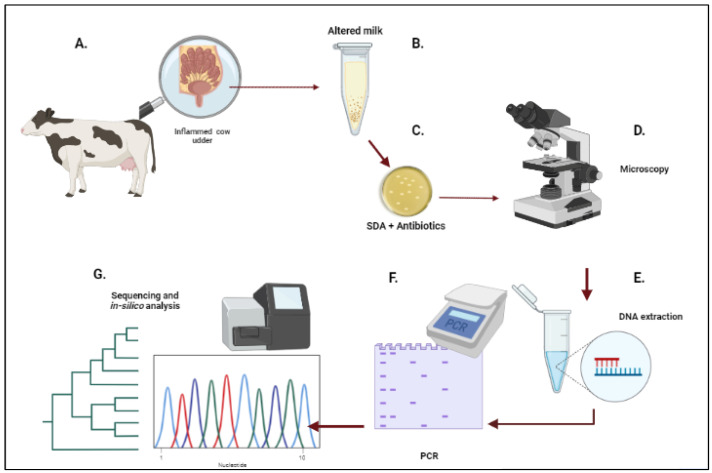
Overview of the methodology described in Materials and Methods. (**A**). Identification of the cow with chronic mastitis. (**B**). Sample collection of milk from the infected cow. (**C**). Microbiological culture of the milk sample on SDA supplemented with vancomycin (5µg/mL) and nalidixic acid (20 µg/mL). (**D**). Microscopy of the samples with visible colony growth and selection of possible *Prototheca* colonies. (**E**). DNA extraction of the samples previously selected with microscopy. (**F**). Molecular identification with endpoint PCR of the *cytB* gene followed by electrophoresis. (**G**). Sequencing of the obtained amplicons (approx. 600 bp) to confirm the identification and in silico analysis of the *Prototheca cytB* sequences. The figure was created with BioRender.com.

From December 2020 to January 2022, milk samples were collected from dairy cows with a previous diagnosis of chronic mastitis. Samples came from six different farms located in a tropical region in the province of Santo Domingo de los Tsáchilas (6 farms with 438 samples), and 20 samples from 3 farms in a much colder climate in the provinces of Pichincha and Tungurahua in the Andean region. Mastitis incidence on these farms at the moment of sampling was no higher than at other times of the year (a non-outbreak situation). The cows’ milk with chronic mastitis had a high somatic cell count (California Mastitis Test (CMT) score of 2–3) and was visibly abnormal. All mastitic cows had clinical signs of udder infection and had previously been treated 2–3 times unsuccessfully with various antibiotics. Milk samples (approx. 10 mL) from the infected quarters were collected in 15 mL conic tubes with caps (one collection vial per cow) after the teats had been washed, dried, and disinfected (with particular attention paid to teat ends) with an iodine solution. The samples were transported at 4 °C to the laboratory for further analysis. In addition, 10 water samples (100 mL), one from each farm, of drinking/washing water for the cows were taken for the isolation of *Prototheca* spp. For an overview of the methodology for the isolation and identification procedure of *Prototheca,* see Figure 1. 

### 2.2. Microbiological Isolation

For the isolation of *Prototheca,* 50 µL of the (now homogenized) milk sample was inoculated in duplicate on Petri dishes with Sabouraud Dextrose Agar (SDA) (BD DifcoTM, Franklin Lakes, NJ, USA) without cycloheximide but supplemented with the antibiotics vancomycin (5 µg/mL) and nalidixic acid (20 µg/mL). For the isolation of *Prototheca* from the water samples, 10 mL were centrifuged in sterile conic tubes with caps at 2500 g for 10 min and the sediment (about 100 µL) was inoculated on the selective plates. Plates were incubated for 5 days at 37 °C.

### 2.3. (Molecular) Identification of Prototheca

**Figure 2:**
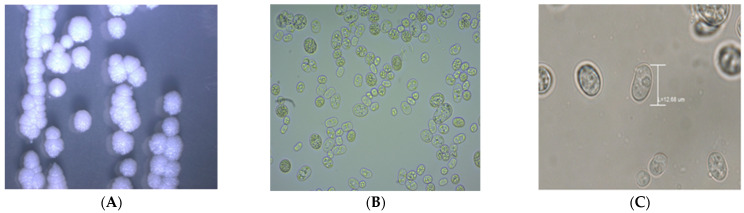
Morphology of *Prototheca bovis* with a light microscope. From left to right: (**A**). Colonies on SDA at 10× magnification. (**B**). A wet mount preparation of *Prototheca* cells at 40× magnification. Some cells show a small amount of green pigment. (**C**). *P. bovis* at 100× magnification. The cells have a diameter of about 12 μm with a thick cell wall and a poorly distinguishable nucleus. The presence of sporangia can be observed. The preparations were photographed using a digital camera (Olympus, Tokyo, Japan) adapted to the light microscope.

Plates with visible growth were inspected with light microscopy to obtain an image at 40x magnification, and photographed using a digital camera (Olympus, Tokyo, Japan) adapted to the microscope (Olympus CX-43, Tokyo, Japan). Additionally, a wet mount preparation of the growth was inspected at 40× and 100× magnification(See Figure 2).Based on this microscopic observation, possible *Prototheca* spp. microorganisms were then harvested from the plate, cryopreserved in SDA Broth (BD DifcoTM, NJ, USA) with 10% glycerol and stored at −20 °C for further analysis. 

To identify the Prototheca-like microorganisms, the Vitek^®^2 System (BioMérieux, Marcy-l’Étoile, France) was used with a YST ID Card for yeast identification [20]. This device is an automated system for the identification and antimicrobial susceptibility of microorganisms. In addition, it was necessary to confirm whether the isolated microorganism visually identified with the light microscope as *Prototheca* spp. belonged to the genus *Prototheca* spp., and to determine the species. Hence, identification was performed with sequence analysis of the mitochondrial cytochrome b (c*ytB*) gene, one of the most commonly used genetic loci in both taxonomy and forensic science for the purposes of species identification [21,22].

*DNA extraction* was performed using the boiling method: suspending 2–3 colonies in 200 μL of Tris EDTA 10/1 (TE 10 mM Tris-HCl pH 8.0 and 1mM EDTA) in a microtube, heating the sample at 100 °C for 10 min, centrifuging at 3500 rpm for three minutes and separating the supernatant. The supernatant that contains the DNA was stored at −20 °C for future use in PCR for identification. 

*Endpoint Polymerase Chain Reaction (PCR)* was performed to amplify 650 bp of the cytochrome B (c*ytB*) gene with primers specific for the genus *Prototheca* [23]: forward primer *cytB*_F1 (5′-GyGTwGAACAyATTATGAGAG-3′) and reverse primer *cytB*_R2 (5′-wACCCATAArAArTACCATTCWGG-3′). A final PCR volume of 25 μL was used containing 12.5 μL Promega GoTaq^®^ DNA Polymerase (Promega, Madison, WI, USA), 0.5 μL (0.2 μM) of *cytB*_F1, 0.5 μL (0.2 μM) of *cytB*_R2, 7.5 μL of Milli-Q ultrapure water and 5.0 μL of DNA. The PCR amplification was performed in an Eppendorf Mastercycler^®^ Gradient thermal cycler (Eppendorf, Hamburg, Germany) with the following cycle conditions: 5 min of initial denaturation at 95 °C, followed by 35 cycles of 30 s at 95 °C, 30 s at 48 °C, and 30 s at 72 °C, and a final extension of 5 min at 72 °C. After electrophoresis, amplification products were visualized on a 2% (*w*/*v*) agarose gel in 1× Tris-borate- EDTA (TBE) buffer with SYBR Safe reagent (Invitrogen, Waltham, MA, USA). 

*Sanger sequencing* was performed for PCR products of approximately 650 bp of the *cytb* gen in an ABI 3500xL Genetic Analyzer (Applied Biosystems, Waltham, MA, USA) with a BigDye Terminator v3.1 Cycle Sequencing Kit.

### 2.4. Bioinformatics and Phylogenetic Analysis

The obtained *cytB* sequences were compared with sequences present in the GenBank databases using the nt-BLAST tool for species identification, and then submitted for the acquisition of an accession number. The sequences were also analyzed in silico using the program MEGA X Molecular Genetic and Evolutionary Analysis Version 10.2.6, along with the Unipro UGENE v41.0 tool. The sequences were used to establish a phylogenetic tree using the maximum likelihood algorithm and the Tamura-Nei model, along with the G+I distribution with a total of 500 Bootstrap. The tree was supplemented with other sequences retrieved from the GenBank database of the *cytB* gene for other *Prototheca* species and *cytB* sequences of the *Auxenochlorella*, *Helicosporidium,* and *Chlorella* genera as outgroups (see Supplementary Table S1). 

### 2.5. Efficacy Testing of Disinfectants

The disinfectants in use on the farms in this study (an organic acid/peroxy acid disinfectant and a chlorine dioxide solution) were tested for their efficacy in the elimination of *Prototheca* with a quantitative suspension test, as has been described in the European Standard EN 14885:2018. Both disinfectants are used on the farms during milking to decontaminate the milking clusters with rubber milking liners after each cow. The working solutions of the disinfectants were prepared according to the manufacturer’s instructions. A dense suspension of a *P. bovis* strain, isolated on one of the farms using aseptic techniques, was prepared in a sterile 0.9% NaCl solution and diluted to obtain a cell concentration of 2–5 × 10 ^6^ cfu/mL. 100 µL of this cell culture was added to 900 µL of the diluted and undiluted disinfectant and incubated for 2 min contact time at room temperature. 100 µL of this solution was then transferred directly to 900 µL of a 10% BSA solution to inactivate the disinfectants. 10 µL of neutralized suspension was spotted on an SDA plate and incubated for 5 days at 37 °C. The number of colonies was counted on the spot to determine the killing rate of the disinfectant. In between the steps of this protocol, a vortex was used to guarantee a homogenous cell suspension.

### 2.6. Ethical Considerations

In Ecuador, there are no animal research committees that evaluate research protocols for animals, and no special permission is needed for this type of study. Moreover, this is a cross-sectional noninvasive diagnostic study, directed to improve the health of the cows. Cattle handling and the collection of the milk samples were carried out by an experienced veterinarian who avoided causing any stress to the animals. Farmers in this study signed a consent form for research, and participation in this study was voluntary. 

## 3. Results

### 3.1. Culture Results and Identification

A total of 458 milk samples from 458 cows with chronic mastitis were obtained and inoculated on SDA plates. Growth that appeared after 5 days of incubation at 37 °C was checked with the light microscope for compatibility with *Prototheca* cells (Figure 2). Only the six different farms located in a tropical region in the province of Santo Domingo de los Tsáchilas (438 samples) yielded 108 *Prototheca*-like microorganisms, while the cultures of the 20 samples from 3 farms from a much colder climate in the Andes were negative. In addition, the water samples were all negative for the growth of *Prototheca*-like microorganisms.

For an initial identification of the *Prototheca*-like microorganisms, we used the Vitek^®^2 System (BioMérieux) with a YST ID Card for yeast identification [18]. The Vitek system identified our isolates as *Prototheca zopfii*. However, recently, *P. zopfii* has been divided into two species (*P. ciferri* and *P. bovis*) [5], thus further differentiation was necessary. All *Prototheca*-like isolates were tested with PCR for the amplification of the *cytB* gene with primers specific for *Prototheca* species. Of the 108 microorganisms, 69 isolates were positive for the *cytB* gene PCR with an amplification product of 644 bp. The other isolates (negative for the *cytB* PCR) that grew on the plates were identified as yeast or fungal species. These were not further identified and their clinical significance is unknown. All 69 PCR products were sequenced, and the sequences were analyzed using the NCBI BLAST platform. This analysis revealed that the PCR product was derived from the *P. bovis cytB sequence*, showing a similarity of 99% with the GenBank *cytB* sequences of *P. bovis*. An overall occurrence of *P. bovis* infection (69/458 cows) in chronic mastitis of 15.1% [11.0–19.6] 95% CI was established.

### 3.2. In Silico Sequence Analysis of the cytB Gene: Intraspecific Variation and Phylogenetic Relationships

All 69 *cytB* sequences were 100% similar and thus cannot be used for an intraspecific variation analysis. Eleven sequences were deposited in GenBank under the accession numbers OM221504–OM221514. For a better confirmation that we had isolated *P. bovis,* a phylogenetic analysis was realized. It showed that our *cytB* sequence belonged to the *Prototheca bovis* group, with a bootstrap value of 98–99 for each branch of species isolated and deposited in GenBank by other countries. The paraphyly of the *P. bovis* sequences and *P. ciferii* sequences is shown. The analysis also established a relationship with the *cytB* sequences of *Helicosporidium* among an entire group of different *Prototheca* species and between *Auxenochlorella* and *Prototheca xanthoriae* sequences, having a branch value of 69 and 89, respectively (see the Supplementary Figure S1 for this phylogenetic tree).

### 3.3. Efficacy Testing of Disinfectants

Theoretically, the 10 µL *Prototheca* suspension, spotted on the plates after incubation with the disinfectant and after the neutralization step, diluted in 10% BSA solution, contained about 2–5 × 10^3^ living *Prototheca bovis* cells. The contact time used in the efficacy testing was 2 min. For the incubations with the undiluted disinfectants, no growth was seen on the plates, demonstrating that all the cells had been killed. After incubating *P. bovis* in the diluted disinfectants, the 10 µL spot showed a confluent growth that was impossible to count, indicating that both diluted disinfectants after 2 min of incubation had minimal or no killing effect on the algal suspension. 

## 4. Discussion

### 4.1. Epidemiology

To our knowledge, this is the first time that *P. bovis* infection has been reported as the cause of chronic mastitis in Ecuador. We found a local prevalence of *Prototheca* infection of approximately 15% in the cows with chronic mastitis, a relatively high occurrence considering that we did not sample in a special epidemiological situation (a sudden increase in mastitis cases). In mastitis outbreak situations, an even higher prevalence can be found with up to 30% of the mastitic cows infected [15,16,17]. All animals positive for Protothecosis in this study were culled, representing considerable economic losses for the farm owners. 

Previously, in Latin America, there have been studies of bovine mastitis caused by *Prototheca* in Brazil, Chile, and Mexico [24,25,26,27,28], evidencing the presence of the pathogen across the South American continent. We isolated *P. bovis* only from samples coming from a tropical area, since the 20 samples from the colder area (the Ecuadorian Andes) were all negative for *Prototheca*. This does not mean that no *Prototheca* infection will be found in the future in the Andes region. In Poland, with its very cold winters, mastitis due to *Prototheca* species is relatively common [29]; indeed, it is the third most common pathogen of bovine mastitis, with an overall incidence of 4.6%.

### 4.2. Isolation of Prototheca on SDA Culture Medium

We isolated *Prototheca* on SDA plates with antibiotics to avoid overgrowth with other bacteria such as *S. aureus,* a fast-growing bacterium, and described it as the lead pathogen causing bovine mastitis infections [30,31]. Two different classes of antibiotics were chosen because these inhibit the growth of most gram-positive and gram-negative bacteria. Fast-growing microorganisms can mask the growth of *Prototheca* species that need 3–5 days to form a visible colony on culture media. Using antibiotics in our culture plates, however, means that no information concerning other microorganisms involved in bovine mastitis was obtained in this study. 

### 4.3. Do We Sub-Diagnose Prototheca Infections in the Microbiology Laboratory?

It is widely accepted that bovine mastitis is mainly bacterial in origin. However, in general, about 20 to 35% of clinical mastitis cases are of unknown etiology because no microorganisms are isolated from the milk of the mastitic cow [32]. Although part of these mastitis cases can have a viral origin, we cannot exclude that in some of these culture-negative cases, the *Prototheca* infection has been missed. We had to culture the milk samples for 4–5 days before visible colonies were seen on the agar plates. In general, microbiological laboratories culture for a maximum of 48 h for the isolation of bacteria, and report culture plates without visible growth as being negative. In doing so, *Prototheca* infections will be missed. For a maximum yield in *Prototheca* recovery, the plates should be kept for at least 5 days in the incubator. Moreover, we inoculated only 50 µL of the milk samples on the selective plates. Growth in 10% of our isolates was limited to only 2–3 colonies. Recovery of *Prototheca* can probably be improved with the introduction of a centrifugation step for the milk sample (2000× *g* for 10 min) and inoculation of the sediment so as to detect low-grade infection. Only in this way will we get a better idea of the real prevalence of *P. bovis* infection. Moreover, a culture plate with antibiotics should always be used for *Prototheca* culturing. Overgrowth of other microorganisms should be avoided, as this can mask the growth of *Prototheca* species. The fact that we missed *Prototheca* infections in the laboratory has been clearly demonstrated in a publication where PCR was used for the detection of *Prototheca* and compared with culture. This study showed 9.3% culture-positive samples versus 16.3% PCR-positive samples, an increase in the detection rate of nearly 100% [33].

### 4.4. Identification of Prototheca Species

Concerning the identification with the light microscope, the morphology of our *P. bovis* was similar to those already described in the literature [1,2]. We observed round or oval cells with a diameter of 12–40 μm, and inside the cell a spheroidal sporangium in which spores are produced (Figure 2). However, using microscopy, we selected 108 look-alike microorganisms, of which only 69 were real *Prototheca* strains. This means that one cannot trust the eye and further identification is always necessary. We used sequencing of the *cytB* gene to identify the *Prototheca* species. Despite being an excellent marker for species identification, the *cytB* gene was inadequate when we tried to use it as a molecular tool for intra-species analysis, as no point mutations were found in the 600 bp of *cytB* of the 69 isolates from this study. 

### 4.5. Phylogenetic Analysis

The sequence data of the *cytB* gen allowed the construction of a phylogenetic tree, and our sequence data formed clusters with other *cytB* gene sequences of *P*. *bovis* strains retrieved from the GenBank database. For this phylogenetic tree, see the Supplementary Figure S1. Each sequence had high bootstrap values per branch of 98 to 99. The tree disposition showed paraphyly of the *Prototheca bovis* sequences and *Prototheca ciferii* sequences. Such phylogenetic configuration confirms the division of the previous clade of the *Prototheca zopffii* genotypes I and II into individual species (*P*. *ciferii* and *P. bovis*, respectively) [5]. Within the tree, a small cluster between *Prototheca xanthoriae* and *Auxenochlorella protothecoides* was formed, having a branch value of 89, thus exhibiting a paraphyletic group of the involved species. Disposition of the branches also displayed a polyphyletic group with the sequences of *Helicosporidium* sp. The association with these two genera (*Auxenochlorella* and *Helicosporidium*) is the same as has been determined in a previous publication [2], *Auxenochlorella* being the closest photosynthetic relative of *Prototheca* and *Helicosporidium* a non-photosynthetic alga with a preference for invertebrates as its main hosts [2,34]. 

### 4.6. Treatment of Prototheca Infection in Bovine Mastitis

For the treatment of *Prototheca* infections in human patients, amphotericin B remains the most effective agent, especially for disseminated *Prototheca* infections. Voriconazole, posaconazole, itraconazole, and fluconazole have also been used successfully in individual cases. Surgery and topical amphotericin B have been used for cutaneous infections [4]. However, for bovine mastitis due to *Prototheca* spp., no treatment currently exists. In vitro tests have shown that some antimycotics, such as amphotericin B and nystatin, have an effect, but these are not frequently used for the treatment of mastitis and are rather expensive [35]. The alga is resistant to all antimicrobials traditionally used in the treatment of mastitis, this being another one of the characteristics that make us suspect an infection by this agent. 

### 4.7. Control of Bovine Mastitis Due to Prototheca

As there is no treatment option, the most practical and effective measure for control is to eliminate infected animals in order to prevent the spread of infection to the rest of the herd. Infected cows are a reservoir of *Prototheca* and, through the milk, a source of infection for other cows. Manure probably plays an important role in the dissemination of *Prototheca* species since the alga can be isolated from the intestine of the cow [36,37]. *Prototheca* mastitis can spread directly from cow to cow at milking time, or indirectly by contamination of the environment. Infected cows should therefore be separated and milked last, preferable with a separate milking unit until they can be removed from the herd [35]. Moreover, mastitis should be detected at an early stage (subclinical) before the symptoms appear with the California Mastitis Test (elevated somatic cell count) and a specific laboratory exam requesting the isolation of *Prototheca.* This can overcome the progress of the *Prototheca* infection into clinical stages, avoiding transmission to other cows and unnecessary antibiotic use. In summarizing, the following recommendations should be considered if confirmed *Prototheca* mastitic cows are found on a farm in order to limit the spread of these microalgae [35].

(1) Identify infected cows through the California Mastitis test and bacteriological analysis with selective medium. (2) Mark infected cows so they can be easily identified and separated from the healthy cows and milked last. (3) Do not attempt the treatment of *Prototheca* infected quarters. Antibiotics do not work on algal infections. (4) Cull *Prototheca* infected cows as soon as it is reasonable to remove them. (5) Good hygiene during milking procedures and hygienic conditions of the milking equipment is important for infection control. The equipment should be disinfected regularly to prevent the infection from spreading further. Moreover, excellent hygiene of both bedding and of aisles/runs should be provided [18,19]. 

### 4.8. Disinfectants and Efficacy for the Elimination of Prototheca spp.

We showed that the disinfectants in the working dilution as recommended by the provider did not eliminate a *Prototheca* inoculum, but the undiluted disinfectant killed the inoculum completely. Both disinfectants were in use for the decontamination of the milking machines and, more specifically, the milking clusters. Possibly, the alga can be transferred from one cow to another and between mammary quarters via the hands of the milkers, or via equipment. 

Poor hygiene of the clusters could be the origin of *Prototheca* infections. For the testing of the disinfectants, we choose a contact time of two minutes. This is a reasonable time, as disinfecting the milking clusters after each cow is a fast process, and in less than 2 min this equipment is connected again to the teats of the next cow. Several other studies have evaluated the efficacy of disinfectants in eliminating *Prototheca*. A study in Poland used three classes of teat disinfectants: an iodine solution, quaternary ammonium compounds, and dodecylbenzenesulfonic acid with 5 min of contact time demonstrated the efficacy of those three classes against *P. zopfii*, with iodine being the most pronounced [38]. Another study evaluated alkyldiaminoethylglycine hydrochloride, chlorhexidine, dioxide chlorine, povidone iodine, and sodium hypochlorous acid against isolates of *P. zopfii* using the microdilution method and incubating the strains for 48 h in the presence of the disinfectant. This in vitro study indicated that all disinfectants except dioxide chlorine were effective against *P. zopfii* [39]. A study in Brazil has shown that low concentrations of sodium hypochlorite and iodine were effective [40]. However, in this study, a contact time of 12 h was used. Another study showed that *P. zopfii* could be isolated from the teat cups [15,33] after being immersed in a chlorine solution. Different contact times and different methodologies used in the cited studies make it impossible to compare, and we cannot recommend a specific disinfectant for the elimination of *Prototheca* species. More studies that outline a well-defined contact time are needed in order to show which disinfectant is effective. It is important to remember that *Prototheca* algae produce spores, and these are normally the most resistant against disinfectants and in disinfection processes. 

### 4.9. Infection Sources of Prototheca

*Prototheca* mastitis is most likely to occur due to contact with contaminated water sources or equipment and once the cow gets infected, *Prototheca* spp. can be spread through the milking process or from cow to calf [8]. The fact that *Prototheca* spp. can survive most standard disinfection processes such as chlorination [40], or are able to survive high temperatures including the pasteurizing temperatures [38], makes cleaning for the elimination of the alga or its spores difficult. Furthermore, the low response against conventional mastitis treatment makes *Prototheca* a mastitis pathogen agent of concern that deserves more attention. There is little information concerning risk factors for infection. Interestingly, a risk factor study in Ontario, Canada concluded that unsanitary or repeated intramammary infusions, antibiotic treatment, and off-label use of injectable drugs in the udder might promote *Prototheca* udder infection, with *Prototheca* acting as an opportunistic pathogen that is promoted by antibiotic-induced suppression of the natural udder flora [41,42].

## 5. Conclusions and Recommendations

This is the first report of chronic bovine mastitis caused by *Prototheca* in Ecuador. We isolated *Prototheca bovis* and determined a high occurrence in dairy cattle with chronic mastitis. It is important to acknowledge that despite the vast number of samples, only *P. bovis* was detected and not *P. blaschkeae*, which is described as another species causing bovine mastitis [14,42]. In a future study, we should determine if *Prototheca* mastitis is a local problem or a general health problem for dairy cows in Ecuador. 

## 6. Study Limitations

This is not a survey on the prevalence of *Prototheca* mastitis in Ecuador, but rather an insight into a local prevalence of *Prototheca* mastitis. Farms that collaborated in the study were limited to a special area of the country, participation in this study was voluntary, no sample calculation was performed, and only chronic mastitis cases were analyzed. Subclinical and acute mastitis were not analyzed for the presence of *Prototheca*. We determined the presence of *Prototheca* in the Andean region, a region with a moderate climate, only with a limited number of samples. In a future study, we should determine if Protothecosis is a local problem or a general health problem for dairy cows in our country. Milk samples were only inoculated in culture media with antibiotics, as we were looking for *Prototheca* spp. We did not look for gram-positive/negative microorganisms as the cause of mastitis, thus no data for co-infection are available. Yeasts and fungi, which occasionally grew on the culture plates, were not identified.

## Data Availability

Not applicable.

## Supplementary Materials

The following supporting information can be downloaded at: https://www.mdpi.com/article/10.3390/vetsci9120659/s1, Figure S1: Phylogenetic tree of *Prototheca* spp.; Table S1: Sequences retrieved from the GenBank used to develop the phylogenetic tree.
