# Supplementary material for: High Prevalence of Prototheca bovis Infection in Dairy Cattle with Chronic Mastitis in Ecuador
Source: Vet Sci. 2022 Nov 25;9(12):659. doi: 10.3390/vetsci9120659 (PMC9784310; doi:10.3390/vetsci9120659)
Supplement: Supplementary file 1 [file vetsci-09-00659-s001.zip › vetsci-1950338-supplementary.pdf]

Supplementary files.

Figure S1. Phylogenetic tree of the *Prototheca* genus built with the MEGA bioinformatics program using the Maximum likelihood algorithm along with the Tamura-Nei model with G+I distribution and bootstrap of 500. Tree results clustered the cytB sequences from this study (seen as "OM#sample-Ecuador" within the *Prototheca bovis* clade.

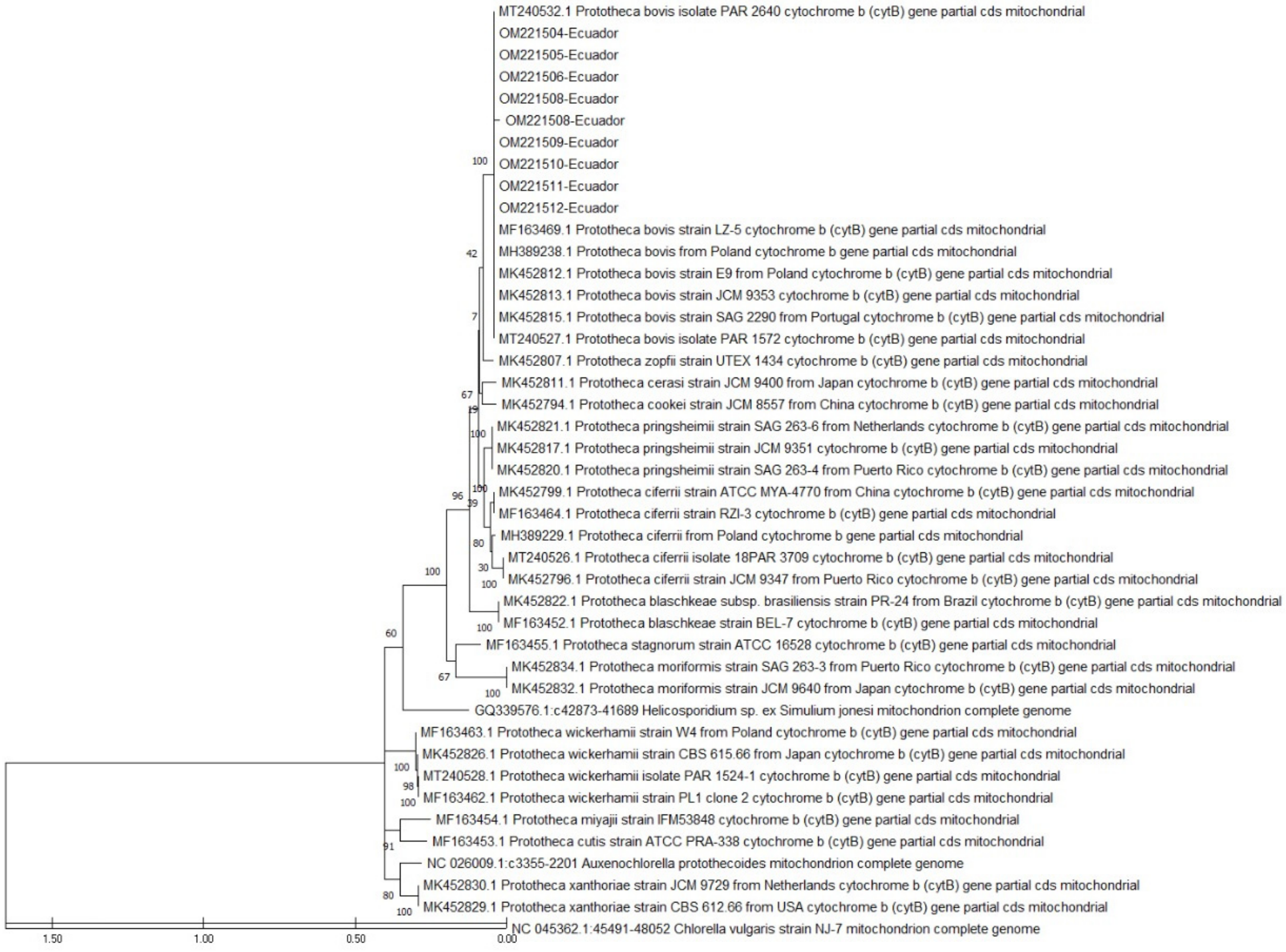

Table S1. Sequences retrieved from the GenBank used to develop the phylogenetic tree of the present study. Table shows the accession number of the sequence together with the species and country of origin.

Accession number	Species	Country
NC_045362.1	Chlorella vulgaris strain NJ-7	China
NC_026009.1	Auxenochlorella protothecoides	China
MT240532.1	Prototheca bovis isolate PAR 2640	Italia
MT240528.1	Prototheca wickerhamii isolate PAR 1524-1	Italia
MT240527.1	Prototheca bovis isolate PAR 1572	Italia
MT240526.1	Prototheca ciferrii isolate 18PAR 3709	Italia
MK452834.1	Prototheca moriformis strain SAG 263-3	Puerto Rico
MK452832.1	Prototheca moriformis strain JCM 9640	Japan
MK452830.1	Prototheca xanthoriae strain JCM 9729	Netherlands
MK452826.1	Prototheca wickerhamii strain CBS 615.66	Japan
MK452822.1	Prototheca blaschkeae subsp. brasiliensis strain PR-24	Brazil
MK452821.1	Prototheca pringsheimii strain SAG 263-6	Netherlands
MK452820.1	Prototheca pringsheimii strain SAG 263-4	Puerto Rico
MK452817.1	Prototheca pringsheimii strain JCM 9351	Poland
MK452815.1	Prototheca bovis strain SAG 2290	Portugal
MK452813.1	Prototheca bovis strain JCM 9353	Poland
MK452812.1	Prototheca bovis strain E9	Poland
MK452811.1	Prototheca cerasi strain JCM 9400	Japan
MK452807.1	Prototheca zopfii strain UTEX 1434	Poland
MK452799.1	Prototheca ciferrii strain ATCC MYA-4770	China
MK452796.1	Prototheca ciferrii strain JCM 9347	Puerto Rico
MK452794.1	Prototheca cookei strain JCM 8557	China
MH389238.1	Prototheca bovis	Poland
MH389229.1	Prototheca ciferrii	Poland
MF163469.1	Prototheca bovis strain LZ-5	Poland
MF163464.1	Prototheca ciferrii strain RZI-3	Poland
MF163463.1	Prototheca wickerhamii strain W4	Poland
MF163462.1	Prototheca wickerhamii strain PL1 clone 2	Poland
MF163455.1	Prototheca stagnorum strain ATCC 16528	Poland
MF163454.1	Prototheca miyajii strain IFM53848	Poland
MF163453.1	Prototheca cutis strain ATCC PRA-338	Poland
MF163452.1	Prototheca blaschkeae strain BEL-7	Poland
GQ339576.1	Helicosporidium sp. ex Simulium jonesi	Canada
